# Cystitis due to the use of ketamine as a recreational drug: a case report

**DOI:** 10.1186/1752-1947-2-219

**Published:** 2008-06-26

**Authors:** Britt Colebunders, Peter Van Erps

**Affiliations:** 1 University of Antwerp, Universiteitsplein, B 2610 Antwerp, Belgium; 2 Department of Urology, ZNA Middelheim, Lindendreef, B 2020 Antwerp, Belgium

## Abstract

**Introduction:**

Ketamine is a derivative of phencyclidine and is a dissociative anaesthetic. Its use as a recreational drug is on the increase among young adults attending clubs and parties.

**Case presentation:**

We describe the case of a 20-year-old man who presented with a 7-month history of urinary frequency, nocturia, urgency, suprapubic discomfort during micturition and episodes of severe haematuria shortly after commencing weekly recreational ketamine use. Complementary examinations were negative except for a thickened bladder wall on ultrasound examination and mild inflammatory changes on cystoscopy. So far only nine cases of ketamine-associated ulcerative cystitis have been described.

**Conclusion:**

We expect that in the future an increasing number of cases of cystitis caused by ketamine use will be seen in young adults.

## Introduction

Ketamine is a derivative of phencyclidine, a popular street drug which is known as 'PCP' or 'angel dust'. Ketamine is less potent and shorter acting compared with phencyclidine and is used as a dissociative anaesthetic in humans [1]. Ketamine, known as 'Special K', is becoming more widely used among young adults attending clubs and parties, including raves [2]. It is labelled a 'club drug' by the National Institute on Drug Abuse (NIDA) of the United States. The effects of ketamine include profound changes in consciousness and psychotomimetic symptoms, such as out-of-body experiences [3]. It can also induce a state of virtual helplessness and a pronounced lack of coordination [4]. Negative effects include increased heart and respiratory rates, nausea and vomiting, convulsions, temporary paralysis and hallucinations [2]. So far only one report has described the effect of ketamine on the urinary system: nine patients were found to have developed a ketamine-associated ulcerative cystitis [5]. We report an additional case.

## Case presentation

We describe the case of a 20-year-old man who presented with a 7-month history of urinary frequency, nocturia, urgency, suprapubic discomfort during micturition and episodes of severe haematuria shortly after commencing weekly recreational ketamine use. The patient occasionally works as a disk jockey at 'hardstyle' and 'jump' parties. His past medical history was significant for nose polyps and asthma, for which he was treated with montelukast (Singulair^®^) and fluticasone propionate in combination with salmeterol (Seretide^®^). He had never travelled outside of Europe.

After 2 months of symptoms he had been treated with antibiotics for 5 days and anticholinergics for several weeks without any improvement. Routine urine analysis and urine cytology were negative and a urine culture was sterile. An ultrasound examination revealed a thickened bladder wall and a small bladder capacity but normal kidneys. Cystoscopy showed mild inflammatory changes, although there was no visual blood in the urine. Bladder biopsies were negative; however, they were not taken during an episode of active cystitis. We advised the patient to stop ketamine use.

## Discussion

Ketamine-associated cystitis appears to be a new clinical entity. So far only nine cases have been described, all of which reported daily ketamine users who presented with severe dysuria, frequency, urgency and severe haematuria [5] (Table 1). Urine cultures were sterile in all patients. Computed tomography revealed marked thickening of the bladder wall, a small bladder capacity and perivesicular stranding, consistent with severe inflammation. At cystoscopy, the bladder walls of eight patients showed multiple erythematous patches. In one patient mild squamous metaplasia and reddened flat ulcerated patches were noted on cystoscopy. Biopsies in four patients revealed epithelial denudation and inflammation with a mild eosinophilic infiltrate. All patients benefited from cessation of ketamine use. In one case the addition of pentosane polysulphate appeared to provide some symptomatic relief.

**Table 1:** Characteristics of 10 patients with ketamine-associated cystitis reported in the literature.

	Our patient	Patient 1	Patient 2	Patient 3	Patients 4–9
**Age**	20 years	28 years	25 years	17 years	Unknown
**Sex**	Man	Man	Woman	Man	Unknown
**Ketamine use**	Weekly	Daily	Daily	Daily	Daily
**Duration of symptoms**	7 months	6 months	2 years	Several months	Unknown
**Urine cultures**	Sterile	Sterile	Sterile	Sterile	Sterile
**Bladder wall**	Thickened	Thickened	Thickened	Thickened	Thickened
**Cystoscopy**	Mild inflammation	Erythematous patches	Mild squamous metaplasia, ulcerated patches	Erythematous patches	Erythematous patches
**Biopsy**	Negative	Epithelial denudation, inflammation, eosinophilic infiltrate	Unknown	Unknown	In three patients, similar to patient 1
**Antibiotic therapy**	Unsuccessful	Unsuccessful	Unknown	Unknown	Unknown
**Benefited from cessation of ketamine**	Unknown	Yes	Yes	Yes	Yes

In our case cystoscopy showed only mild signs of inflammation and biopsies were negative. However, our patient used ketamine only on a weekly basis, whereas the patients described in the literature were daily users. This could explain the difference between our patient's cystoscopy and biopsy findings with those of the nine cases reported in the literature. Moreover, in our patient the biopsies were not taken during an episode of active cystitis. We suspect, however, that ketamine was the cause of the patient's complaints, as the timing of the onset of symptoms correlated strongly with the commencement of ketamine use. In addition, the evidence shows our case to be consistent in many ways with the nine other cases described in the literature (Table 1).

The mechanism by which ketamine induces cystitis is not clear. Ketamine and its metabolites norketamine and hydroxynorketamine can be measured in high quantities in the urine of patients using ketamine [6]. It is possible that ketamine and its active metabolites cause significant bladder irritation.

## Conclusion

As ketamine is being used increasingly as a recreational drug we expect ketamine-associated cystitis to become more prevalent in young adults. Health care workers should be aware of the problem and patients should be informed about the possible side effects of ketamine. The long-term sequelae of ketamine on the bladder remain unknown.

## Competing interests

The authors declare that they have no competing interests.

## Consent

Written informed consent was obtained from the patient for publication of this case report and any accompanying images. A copy of the written consent is available for review by the Editor-in-Chief of this journal.

## Authors' contributions

BC reviewed the literature, and conceived of and drafted the manuscript, PVE is the department chair, who provided general support. Both authors revised and approved the manuscript.
